# How does feedback from phage infections influence the evolution of phase variation in *Campylobacter*?

**DOI:** 10.1371/journal.pcbi.1009067

**Published:** 2021-06-14

**Authors:** Simran K. Sandhu, Christopher D. Bayliss, Andrew Yu. Morozov

**Affiliations:** 1 Department of Mathematics, University of Leicester, Leicester, United Kingdom; 2 Department of Genetics and Genome Biology, University of Leicester, Leicester, United Kingdom; 3 Institute of Ecology and Evolution, Russian Academy of Sciences, Moscow, Russia; University of Florida, UNITED STATES

## Abstract

*Campylobacter jejuni* (*C. jejuni*) causes gastroenteritis following the consumption of contaminated poultry meat, resulting in a large health and economic burden worldwide. Phage therapy is a promising technique for eradicating *C. jejuni* from poultry flocks and chicken carcasses. However, *C. jejuni* can resist infections by some phages through stochastic, phase-variable ON/OFF switching of the phage receptors mediated by simple sequence repeats (SSR). While selection strength and exposure time influence the evolution of SSR-mediated phase variation (PV), phages offer a more complex evolutionary environment as phage replication depends on having a permissive host organism. Here, we build and explore several continuous culture bacteria-phage computational models, each analysing different phase-variable scenarios calibrated to the experimental SSR rates of *C. jejuni* loci and replication parameters for the F336 phage. We simulate the evolution of PV rates via the adaptive dynamics framework for varying levels of selective pressures that act on the phage-resistant state. Our results indicate that growth reducing counter-selection on a single PV locus results in the stable maintenance of the phage, while compensatory selection between bacterial states affects the evolutionary stable mutation rates (i.e. very high and very low mutation rates are evolutionarily disadvantageous), whereas, in the absence of either selective pressure the evolution of PV rates results in mutation rates below the basal values. Contrastingly, a biologically-relevant model with two phase-variable loci resulted in phage extinction and locking of the bacteria into a phage-resistant state suggesting that another counter-selective pressure is required, instance, the use of a distinct phage whose receptor is an F336-phage-resistant state. We conclude that a delicate balance between counter-selection and phage-attack can result in both the evolution of phase-variable phage receptors and persistence of PV-receptor-specific phage.

## Author summary

Globally rising rates of antibiotic resistance have renewed interest in phage therapy. Bacteriophages (phages) act on bacteria to select for resistance mechanisms such as loss of phage receptors by phase variation (PV). Phase-variable genes mediate rapid adaption by stochastic switching of gene expression. *Campylobacter jejuni* is a common commensal of birds but also causes serious gastrointestinal infections in humans. Optimisation of phage therapy against *C. jejuni* requires an in-depth understanding of how PV has evolved and mediates phage resistance. Here, we use a detailed continuous culture model for nutrient-limited bacteria-phage interactions, with PV rates calibrated to match the experimental observations for *C.jejuni* and phage F336. Evolution within a model accounting for two phase-variable loci closely matches the experimental results when growth reducing counter-selection is imposed on all phage-resistant states, but, not when restricted to the particular states associated with resistance to immune effectors. Our results emphasize that delicate balancing of selective pressures, imposed by single and multiple distinct phages, are necessary for effective use of phage therapy against *C. jejuni*.

## 1 Introduction

A major current issue in modern medicine is increasing pathogenic bacterial resistance to available antibiotics, this concern has resulted in the development of alternative bacteria-fighting techniques becoming one of the highest priorities of modern medicine [1]. One long-standing but under-explored alternative is bacteriophage (or simply phage) therapy. Bacteriophages are viruses that infect bacteria by attachment to specific host receptors, triggering a process of cell invasion that can lead to phage replication (or, if it is a lysogenic phage, invasion of the bacterial chromosome [2, 3]). One approach is to harness the bactericidal activity of lytic phages for treating bacterial infections or in food sciences to prevent meat contamination with unwanted pathogens [4]. Although widely utilised in Eastern Europe, phage therapies are not licensed for treating human or animal infections due to concerns about efficacy and rapid development of bacterial resistance. The use of phages to eradicate Campylobacters from poultry flocks has been promoted as a realistic alternative to limited effect barrier policies or novel vaccines. A key barrier to widespread phage use against Campylobacters is the development of resistance [5, 6]. In this article, we explore the evolution of hypermutable loci as a phage resistance mechanism under dynamic feedback from the abundance of a specific phage and discuss how this resistance mechanism might be circumvented.

*Campylobacter jejuni* is a Gram-negative, spiral-shaped bacterium commonly found as a commensal in the gastrointestinal tract of many species but in particular, birds [7, 8]. *C. jejuni* can cause serious gastroenteritis in humans with a sub-set of these infections triggering pathological autoimmune responses [9–11]. Contaminated chicken meat is a principal source of *C. jejuni* infections for humans and is responsible for a large health and economic burden worldwide causing over 500,000 cases of gastroenteritis every year in the UK alone [12]. Phage therapy is a promising technique for eradicating *C. jejuni* from chicken carcasses and poultry flocks. However, bacterial resistance can develop rapidly over time as phage are dependent on bacteria to multiply and hence phage and their host bacteria are involved in a constant arms race of co-evolution to ensure survival [13, 14].

In several important bacterial pathogens, phase variation (PV) can change the expression states of surface molecules and consequently alter interactions with external factors [15–18]. Simple Sequence Repeats (SSR) tracts are a major mechanism of PV due to a high frequency of variations in repeat number that are thought to occur during DNA replication as a result of slipped-strand mispairing [19]. *C. jejuni* strains contain 12–29 genes with hypermutable homopolymeric G or C tracts of lengths between G7 and G13 (e.g. a G7 tract consists of seven consecutive G bases) [8, 10, 20]. The high mutation rates of these homopolymeric sequences are associated with insertions or deletions of a single base but with a bias towards insertions in G8 and G9 tracts and deletions in G10 and G11 tracts [21–26]. Similar biases, due to different rates of insertions and deletions, have also been detected in other bacterial systems [22–26]. These changes in repeat number produce ON and OFF switches in the expression of the repeat-containing gene, typically by enabling or disabling the translation of a functional gene product [27]. This PV mechanism is not randomly located within Campylobacter genomes but is associated with genes involved in modifications of surface molecules with influence on adaptation to external selective agents [28, 29].

Poultry flocks are the major source of foodborne Campylobacter gastrointestinal infections. Treating birds with phage has been largely unsuccessful due in part to the rapid development of resistance by alterations in the structure or expression of receptors. PV of two genes (*cj1421* and *cj1422*) in *C. jejuni* strain NCTC11168 leads to complete resistance to infection by bacteriophage F336. Both genes add phosphoramidite moieties to the capsular polysaccharide but to different receptor sugars [5, 6]. F336 only binds to bacterial cells when *cj1421* is switched ON and is blocked if *cj1422* is also switched ON or if *cj1421* is switched OFF. Whilst this example shows how reversible variation processes [30] can switch the bacterium between phage resistant and susceptible states, there are still questions about how these switches in expression impact other fitness advantages associated with the susceptible and resistant states [27]. Another study has shown that the phosphoramidite moieties are associated with resistance to killing by serum indicating that there is counter-selection for one of the phage-susceptible states [6]. Two key outstanding questions are why these genes do not become fixed into the phage resistant expression states and if two phase-variable genes are more evolutionarily stable than one. One approach to answering these questions is through computational modelling. This approach has been successfully applied to a variety of bacteria-phage interactions including phage-mediated biodiversity [31, 32], phage-bacteria co-evolution with bacterial resistance [13, 33], control of disease such as cholera outbreaks [34], applications of phage therapy [34–36] and the influence of environmental factors on control of bacteria by phages [37–39].

In this article, we explore the bacteria-phage dynamics within a continuous culture system by building a series of mathematical models [37] each with a differing mutational framework of PV within *C. jejuni*; a simple PV model of switching between susceptible and resistant states [40–42]; a single evolvable PV locus; and two evolvable PV loci. We uncover the evolutionarily stable mutation rates for each scenario and how the ESS is impacted by varying pressure from the phage and bacterial growth reduction. We show that very high and very low mutation rates are evolutionarily disadvantageous. We find that in the system with two phase-variable loci, the absence of either selective force or exertion of high counter-selection can either result in phage extinction or forcing mutation rates to evolve below the basal switching rate. We observe that increasing the complexity of the PV mutation framework results in a lower selective cut-off and evolution of mutation rates close to observed rates [21].

## 2 Materials and methods

### 2.1 Model equations

**Fig 1:**
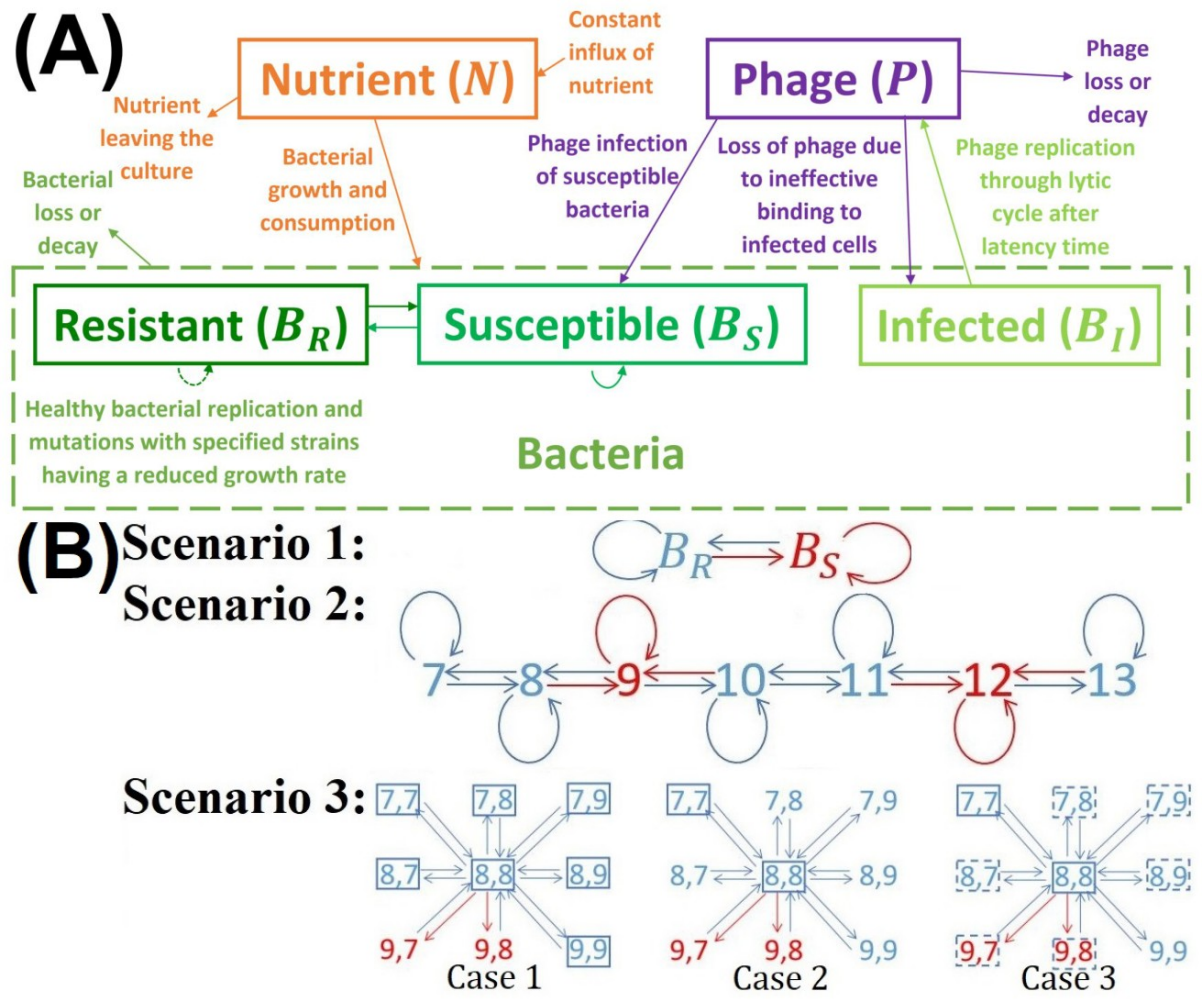
Schematic representations of general model frameworks used to describe the nutrient-phage dynamics within a continuous culture system. (A) Illustrative representation of all interactions accounted for in the model with the following components; growth-limiting nutrient (*N*), phage-resistant bacteria (*B*_*R*_), susceptible phage-free bacteria (*B*_*S*_), infected bacteria in the lytic state (*B*_*I*_) and free phage (*P*). (B) Three scenarios of mutational switches in the expression of the phage receptor (i.e. Phase Variation) that lead to oscillations between phage-resistant and phage-susceptible bacteria. Scenario 1 depicts simple ON and OFF switching of the receptor. Scenario 2 shows evolvable switching due to a polyG tract located in the reading frame of the phage receptor gene. Scenario 3 illustrates the two locus phenomenon where switching ON of gene 2 can block phage binding even when gene 1 is in the ON phage-susceptible state (see text for the biological context). For the two loci scenario, we show, for brevity, a particular example of the interactions of the state with both loci starting with a repeat number of 8. In the actual model, switching extends to all possible states for repeats numbers between 7 and 12. The red states are considered to be phage-susceptible and blue states are phage-resistant while boxes indicate states subject to high (solid line) or medium (dashed line) levels of growth reducing counter-selection. All model components are defined in Table 1.

Our model simulates bacteria-phage interactions with a limited nutrient influx [43] in a continuous culture system, using a conventional delay differential equation modelling approach (used more frequently than the ordinary differential equations framework in the literature [35, 37, 38, 44–46]) as summarised in Fig 1A and using the growth parameters set out in Table 1. Modelling the life cycle of infection, replication and lysis in phage and bacterial populations involves developing and solving expressions for the concentrations of five main components; a growth-limiting nutrient (*N*), resistant bacteria (*B*_*R*_), susceptible phage-free bacteria (*B*_*S*_), infected bacteria in the lytic state (*B*_*I*_) and free phage (*P*) [38]. The general model used is given by;

$$\begin{array}{c} \frac{dN}{dt}=D\left(N_{0}-N\right)-\sum B\frac{\mu_{max}N}{k_{s}+N}\frac{1}{Y}, \end{array}$$ (1)

$$\begin{array}{c} \frac{dB_{R}}{dt}=M\left(B_{R},\epsilon \right)\frac{\mu_{max}N}{k_{s}+N}\left(1-\sigma \right)-B_{R}\left(D+d_{B}\right), \end{array}$$ (2)

$$\begin{array}{c} \frac{dB_{s}}{dt}=M\left(B_{s},\epsilon \right)\frac{\mu_{max}N}{k_{s}+N}-B_{s}\left(D+d_{B}\right)-B_{s}KP, \end{array}$$ (3)

$$\begin{array}{c} \frac{dB_{I}}{dt}=B_{s}KP-e^{-(D+d_{B})T}KB_{s}\left(t-T\right)P\left(t-T\right)-\left(D+d_{B}\right)B_{I}, \end{array}$$ (4)

$$\begin{array}{c} \frac{dP}{dt}=b*e^{-(D+d_{B})T}KB_{s}\left(t-T\right)P\left(t-T\right)-K\left(B_{s}+B_{I}\right)P-\left(D+d_{P}\right)P, \end{array}$$ (5)

where *M* is some function (in general, a matrix function) that accounts for mutational switching between bacterial variants due to PV. These mutational switches occur stochastically as the bacterial cells replicate and hence mutants (or more properly phase variants) are continuously generated during the growth of the bacterial populations. *M* will vary dependent on the mutation scenario; simple PV switching, one and two-loci being subjected to PV, each of these functions are formulated in section 2.2 and summarised in Fig 1B. *M* is dependent on the evolutionary ‘scaling factor’ parameter *ϵ* that is employed to simultaneously modify the magnitude of all mutational switching rates for a specific scenario (e.g. *S* and *R* for scenario 1).

**Table 1:** Definitions of model variables, functions and parameters as well as their default values.

Component	Meaning	Values and Units
*ϵ*	Magnitude of all mutational switching	no units, wide variation range
*μ*_*max*_	Maximal growth rate of phage-free bacteria	$\frac{log(2)}{ReplicationTime}=\frac{log(2)}{0.5}\approx 1.3863$ h^-1^
*d*	Constant background mortality of all cells (rate of decay)	*d*_*b*_ = 0.02 and *d*_*p*_ = 0.05 h^-1^ [53]
*D*	Dilution (wash-out) rate of all cells from the culture	0.2 h^-1^ [48]
*N*_0_	Concentration of nutrient in the influent	30 *μ*g/ml
*Y*	Bacterial yield (bacterial cells per nutrient)	7.4 * 10^4^
*k*_*s*_	Saturation constant (half velocity) of bacterial cells consumption of the nutrient	4 *μ*g/ml
*K*	Phage adsorption rate [51]	1 * 10^−8^ ml/h
*b*	Virus replication factor (burst size)	70
*T*	Lysis time of infected bacterial cells (Latency time)	0.2 h
*σ*	Reduction in fitness of resistant bacteria (or other specified cells) with respect to that of susceptible bacterial cells	0.05
*S*, *R*	Bacterial mutation rate (*ϵ* = 1) from the susceptible state into the resistant state (*S*) and vice versa (*R*), respectively (for the simple switching scenario)	*S* = 0.001, *R* = 0.0018

The nutrient *N*, has a constant influx of *N*_0_ and it is consumed at a rate dependent on the entire bacterial concentration (*B* = *B*_*R*_ + *B*_*S*_ + *B*_*I*_). The continuous growth of all phage-free bacteria (we assume that once infected bacterial cells lose the ability to reproduce) described by a standard logistic function parameterisation [38, 47], where *μ*_*max*_ *N* is their maximal per capita growth rate and *K*_*s*_ + *N* is the carrying capacity of the system. The development of bacterial resistance to phage infections can often result in a selective disadvantage, such as a slower growth rate as compared to the susceptible bacterial strains or higher sensitivity to other selective factors (e.g. immune effector or another phage) [40, 48]. Hence, we introduce some selection against phage-resistant bacteria *B*_*R*_, through the introduction of the parameter *σ* which shows a reduction of 1 − *σ* in the growth rate and fitness of resistant bacteria with respect to that of susceptible bacteria. From here onwards we refer to this growth reducing selection by the term counter-selection in order to emphasize that selection against phage-resistant bacteria (note that in the two gene model selection is differentially applied to each of the multiple phage-resistant variants) could be due to reduced growth characteristics or result from enhanced susceptibility to an alternate selection pressure acting specifically on this phase state. For simplicity, we assume that counter-selection is constant in any scenario where this parameter is applied.

The density of free-phage in the system decreases due to binding to phage-free susceptible bacteria (described by term *B*_*S*_ *KP*) and infected bacteria (with the loss of phage due to ineffective binding described by the term *KB*_*I*_ *P*). The attachment of phages to bacteria and the subsequent infection is commonly assumed to follow mass action kinetics where the rate of infection is assumed to be directly proportional to the concentration of phage and bacteria [44, 48–50]. The density of these free-phage increase once the infected cells complete the lysis cycle. Each infected bacterial cell releases a fixed constant number of new phage particles equal to the burst size *b* [40, 43, 44, 46, 48, 49, 51]. Due to the lysis time of infected cells (latency time) between infection and phage reproduction, we introduce a time delay *T* before newly produced phage particles are released into the culture system [52]. Therefore, the density of infected cells currently undergoing lysis is described by the delay term $KB_{S}(t-T)P(t-T)e^{-(D+d_{B})T}$, where the exponential term accounts for the loss of any infected cells during the lysis process [44, 48]. Hence, the density of free-phage released at the end of the lysis cycle is described by $bKB_{S}(t-T)P(t-T)e^{-(D+d_{B})T}$.

Finally, we also allow for some background mortality (or natural decay) and dilution from the culture of both bacterial and phage cells, the rate at which these cells are lost through death or removal from the system is given by *B*(*D* + *d*_*B*_) and (*D* + *d*_*P*_)*P*, respectively. Phage decay can be spontaneous or alternatively can occur due to severe environmental changes, strong absorption to other surfaces other than bacterial cells or consumption by flagellates [53]. Bacterial cells are far more stable but can still suffer decay when the nutrient is limited or through external predation [54].

In the model, densities of bacterial cells and phage particles are measured in cells or particles/ml. Essential kinetic parameters that characterise the interactions between bacteria and phage are the latency time *T*, burst size *b* and the adsorption rate *K*. Values for these parameters were derived from the existing literature along with some preliminary model simulations (Table 1; [48, 51, 53]). Burst size *b* and the latency time *T* were assumed to be constant within a single simulation, consistent with the limited variation in these parameters described elsewhere [55]. Within our model, a few fundamental assumptions are made. We simplify the evolutionary problem by considering a continuous culture system that removes fluctuations in the nutrient and subsequent frequent fluctuations in bacterial population size. Realistically, bacterial cells have multiple binding sites for the phage to attach and infect, allowing more than a single phage to be absorbed by any given susceptible bacterial cell [50, 56]. Modelling of multiple attachment sites found that the number of binding sites on any given bacterial cell had no significant influence on the concentrations of free phage [46]. As such, we assume that each bacterial cell has an infinite number of attachment sites for free phage but that only one phage will inject genetic material into the cell [20]. For simplicity, we assume that phage always enters the lytic cycle [38] and that there was no lysogenic stage. This assumption is realistic for a subset of phage including F336, which is a lytic phage.

We investigate the evolution of the magnitude of mutation rates, *ϵ*, of phase-variable genes for the *C.jejuni* bacteria during selection from a phage, F336, and with a variable growth reducing counter-selection against the phage resistant state. We explore the impact of these opposing and variable selection pressures on evolutionary stable mutation rates in an adaptive dynamics framework that considers the long-term evolutionary outcome of invasion by a rare bacterial strain with different mutational rates [57, 58]. According to adaptive dynamics, a successful invasion of a mutant strain results in the rare bacterial strain displacing the resident strain. Through an iterative series of invasions and substitutions, the population evolves to an evolutionary stable singular point (ESS) that should be both evolutionarily stable, nearby mutants are not able to invade, and convergent stable, ensuring that the ESS can be attained. We use direct numerical simulations of model equations (see below) to find the outcome of competition between the mutant and the resident strains. In particular, we investigate the ESSs dependence on the strength of growth-reducing selection (described by the parameter *σ*) acting against the phage-resistant variants of bacteria for each of three different PV scenarios that differ in the evolvability of the switching rate and the number of loci (see Fig 1B). In the next section, we explain in detail how mutation between various bacterial states was modelled.

### 2.2 Modelling mutation scenarios

Within this study, three different mutation scenarios were considered; simple switching, one and two loci being subjected to PV. Here we explain in detail how each of these three scenarios was modelled by defining their mutation function *M* within the general model.

For simplicity and ease of notation, we represent the density of all bacterial cells with tract lengths *i* + 6, *j* + 6, *k* + 6 for each of the three loci capable of PV by *b*_*i*,*j*,*k*_ (for the sake of simplicity if any of the loci are not PV capable then their subscript will be dropped). Hence the matrix *B* = {*b*_*i*,*j*,*k*_}_*i*,*j*,*k*=1:7_ represents the concentrations of bacterial cells with all possible tract length combinations.

#### 2.2.1 Scenario 1: Simple susceptible-resistant switching

For this simple switching mechanism we can define the mutation function as *M*(*B*, *ϵ*) = *M*^1^(*ϵ*)*B*, with *B* = [*B*_*R*_ *B*_*S*_] and *M*^1^ defined as follows;

$$M^{1}=[\begin{array}{cc} 1-\epsilon R & \epsilon S\\ \epsilon R & 1-\epsilon S \end{array}]$$ (6)

where *ϵ* is the coefficient determining the magnitude of the mutational switching, which is the evolutionary parameter. We multiply *ϵ* by the experimentally-based mutation rates, *S* and *R* (see Table 1), that determine switching from the resistant to susceptible states, respectively. Note that these rates are identical to the insertion and deletion rates for repeat tracts of lengths 9 and 10, respectively (see Table 2), with 9 being associated with expression of the phage receptor and hence representing the susceptible state whereas 10 lacks the receptor and hence is phage resistant.

**Table 2:** Rates of change in the poly G/ poly C repeat length of phase variable loci within *C. jejuni* upon bacterial replication. The rates of change in tract length, per division, for each tract length, were derived from the probabilities of an insertion or deletion of a poly G/ poly C repeat tract, as given by the experimental data presented in [21].

Tract Length	Insertion Rate (*α*)	Deletion Rate (*γ*)
7	0.0001	0
8	0.0004	0.00004
9	0.001	0.0002
10	0.0004	0.0018
11	0.0002	0.0028
12	0.0001	0.003
13	0	0.004

#### 2.2.2 Observed mutation rates in phase variation in bacteria

Evolvable switching by changes in repeat number allows for shifts in the mutation rate of each switch direction of a phase-variable locus. In *C.jejuni*, PV is mediated by insertions and deletions in poly G / poly C repeat tracts [28, 59] of 7 to 13 repeats with only specific repeats numbers in each tract being associated with an ON or OFF state [15]. Here, we consider PV loci where 9G and 12G are defined as ON and susceptible (*B*_*S*_) to infection while all other lengths are defined as OFF and resistant (*B*_*R*_) to infection. The probabilities of insertions and deletions for each tract length are shown in Table 2 as derived from published observations of PV in *C. jejuni* [15]. For modelling, the tract lengths were restricted to be between 7 and 13 by setting the deletion rate for 7 and the insertion rate for 13 to be 0, that is, the probability for mutating outside these ranges is 0. Tract lengths larger than 13 are rare whereas tract lengths lower than 7 are not usually considered to be phase variable due to low mutation rates (7Gs is the cutoff chosen in the existing literature) [21, 60, 61]. When looking at lower mutation rates we arbitrarily assume that the indel patterns are maintained.

#### 2.2.3 Scenario 2: Single gene PV

This scenario considers only one gene being capable to undergo phase variation, for each tract length *b*_*i*_ there can be potential losses due to insertions or deletions to neighbouring tract lengths, this would occur at a rate (*ϵα*_*i*_ + *ϵγ*_*i*_)*b*_*i*_ (here the evolutionary parameter *ϵ* has the same meaning as in Scenario 1). There can also be an increase in the density of *b*_*i*_ for neighbouring tract lengths mutating, occurring at a rate *ϵα*_*i*−1_ *b*_*i*−1_ + *ϵγ*_*i* + 1_ *b*_*i*+1_. Therefore, the change in *b*_*i*_ due to mutations is described by *b*_*i*_ = *ϵα*_*i*−1_ *b*_*i*−1_ + *ϵγ*_*i*+1_ *b*_*i*+1_ + (1 − *ϵα*_*i*_ − *ϵγ*_*i*_)*b*_*i*_. Therefore, for this scenario the mutation function *M* can be defined by a tridiagonal transition matrix *M*(*B*, *ϵ*) = *M*^2^(*ϵ*)*B*, where the *i*^*th*^ row of *M*^2^ is defined as follows;

$$\begin{array}{c} M_{i}^{2}=[\begin{array}{ccccccccc} 0 & \cdots & 0 & \epsilon \alpha_{i-1} & 1-\epsilon \alpha_{i}-\epsilon \gamma_{i} & \epsilon \gamma_{i+1} & 0 & \cdots & 0 \end{array}] \end{array}$$

where *ϵ* is the magnitude of the mutational switching, *α*_*i*_ is the rate of insertions and *γ*_*i*_ is the rate of deletions for a gene with tract length *i* + 1 given by Table 2.

#### 2.2.4 Scenario 3: Two genes PV

Finally, we consider the more complex scenario where two genes are subjected to phase variation. For this scenario, we have a far more intricate mutation scheme, for each bacteria *b*_*i*,*j*_, all possible mutations are displayed in Fig 1. Using this scheme, we can determine the change of concentration *b*_*i*,*j*_ after mutational switching caused by PV has taken place, this can be described as follows;

$$\begin{array}{c} b_{i,j}=\left(\epsilon \alpha_{i-1}b_{i-1,j}+\epsilon \gamma_{i+1}b_{i+1,j}\right)\theta_{j}\left(\epsilon \right)+\left(\epsilon \alpha_{j-1}b_{i,j-1}+\epsilon \gamma_{j+1}b_{i,j+1}\right)\theta_{i}\left(\epsilon \right)+\epsilon^{2}\alpha_{i-1}\alpha_{j-1}b_{i-1,j-1}\\ \;+\epsilon^{2}\alpha_{i-1}\gamma_{j+1}b_{i-1,j+1}+\epsilon^{2}\gamma_{i+1}\gamma_{j+1}b_{i+1,j+1}+\epsilon^{2}\gamma_{i+1}\alpha_{j-1}b_{i+1,j-1}\\ \;+\left(1-\left(\epsilon \alpha_{i}+\epsilon \gamma_{i}+\epsilon \alpha_{j}+\epsilon \gamma_{j}\right)+\left(\epsilon \alpha_{i}+\epsilon \gamma_{i}\right)\left(\epsilon \alpha_{j}+\epsilon \gamma_{j}\right)\right)b_{i,j}, \end{array}$$

where *θ*(*ϵ*) = 1 − *ϵα* − *ϵγ* is the probability that a bacterial cell’s tract length will not change one of it’s gene tract lengths (the evolutionary parameter *ϵ* has the same meaning as before). Therefore, we can express our mutation function *M* in matrix form through three tridiagonal matrices;

$$\begin{array}{c} M_{i,j,1}^{3}=[\begin{array}{ccccccccc} 0 & \cdots & 0 & \theta \left(\epsilon \right)\epsilon \alpha_{i-1} & 1-U & \theta_{j}\left(\epsilon \right)\epsilon \gamma_{i+1} & 0 & \cdots & 0 \end{array}], \end{array}$$

$$\begin{array}{c} M_{i,j,2}^{3}=[\begin{array}{ccccccccc} 0 & \cdots & 0 & \epsilon^{2}\alpha_{i-1}\alpha_{j-1} & \theta_{i}\left(\epsilon \right)\epsilon \alpha_{j-1} & \epsilon^{2}\gamma_{i+1}\alpha_{j-1} & 0 & \cdots & 0 \end{array}], \end{array}$$

$$\begin{array}{c} M_{i,j,3}^{3}=[\begin{array}{ccccccccc} 0 & \cdots & 0 & \epsilon^{2}\alpha_{i-1}\gamma_{j+1} & \theta_{i}\left(\epsilon \right)\epsilon \gamma_{j+1} & \epsilon^{2}\gamma_{i+1}\gamma_{j+1} & 0 & \cdots & 0 \end{array}], \end{array}$$

where *U* = *ϵ*(*α*_*i*_ + *γ*_*i*_ + *α*_*j*_ + *γ*_*j*_) + *ϵ*^2^(*α*_*i*_ + *γ*_*i*_)(*α*_*j*_ + *γ*_*j*_) and we define the new bacterial concentrations as follows;

$$\begin{array}{c} M\left(b\left(:,j\right),\epsilon \right)=M_{:,j,1}^{3}\left(\epsilon \right)b\left(:,j\right)+M_{:,j,2}^{3}\left(\epsilon \right)b\left(:,j-1\right)+M_{:,j,3}^{3}\left(\epsilon \right)b\left(:,j+1\right) \end{array}$$

for each *j*.

## 3 Results

### 3.1 Scenario 1: Simple susceptible resistant switching

**Fig 2:**
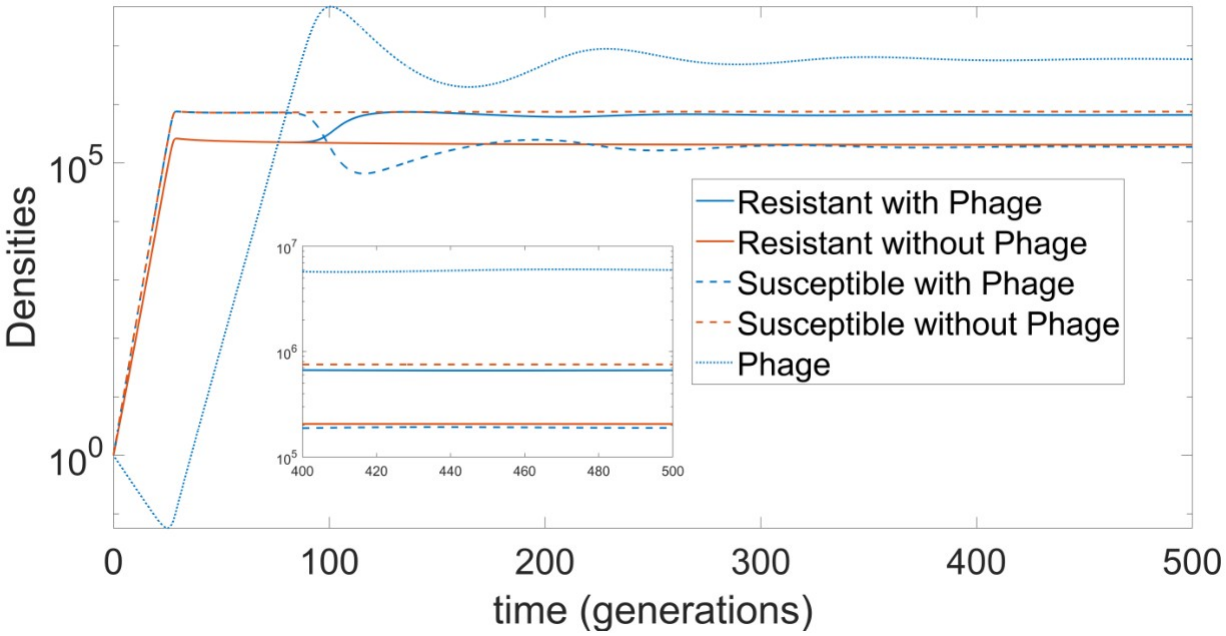
Bacterial and phage densities for the simple switching model (measured in CFU/ml and PFU/ml). The model is described by Eqs (1)–(5) with counter-selection (*σ* = 0.05) and involves a mutation strategy of simple switching between susceptible (*B*_*S*_) and resistant states (*B*_*R*_) at rate of *S* = 0.001 for *B*_*S*_-to-*B*_*R*_ and *R* = 0.0018 for *B*_*R*_-to-*B*_*S*_ (i.e. the scaling factor *ϵ* is set to 1). All other parameter values are taken from Table 1. We show the results for this model in the absence (Model 1, orange curves) or presence (Model 2, blue curves) of phage. Dashed curves, susceptible bacteria; solid curves, resistant bacteria; dotted curves, phage.

We start with the simple PV-based mutation scenario of switching between phage resistant and susceptible states at fixed rates. Similar scenarios have been studied for other bacterial species such as *E. coli* and its predation by bacteriophage T4 [40–42]. The switching rates represent scaled rates for a phase-variable gene in *C. jejuni* with a 1.8-fold higher rate for Resistant-to-Susceptible (*R*) than Susceptible-to-Resistant (*S*). In the absence of phage, the system approaches a steady-state (with very small amplitude oscillations around the state) with a 10-fold higher level of susceptible than resistant bacterial cells (dashed and solid orange lines, respectively; Fig 2).

Addition of phage into the system results in an inversion of the resistant and susceptible concentrations along with a relatively minute reduction in the total bacterial concentration (blue lines, Fig 2), the phage particles are maintained at a 10-fold higher level than the bacterial cells (dotted blue curve). A similar result is observed for the other two mutation scenarios (described below), however, we omit their dynamics for brevity. Note that for the considered values of parameters, the observed pattern of long-term dynamics are very small oscillations of species densities around a stationary state: a further increase of delay time results in the extinction of phage for all three scenarios.

**Fig 3:**
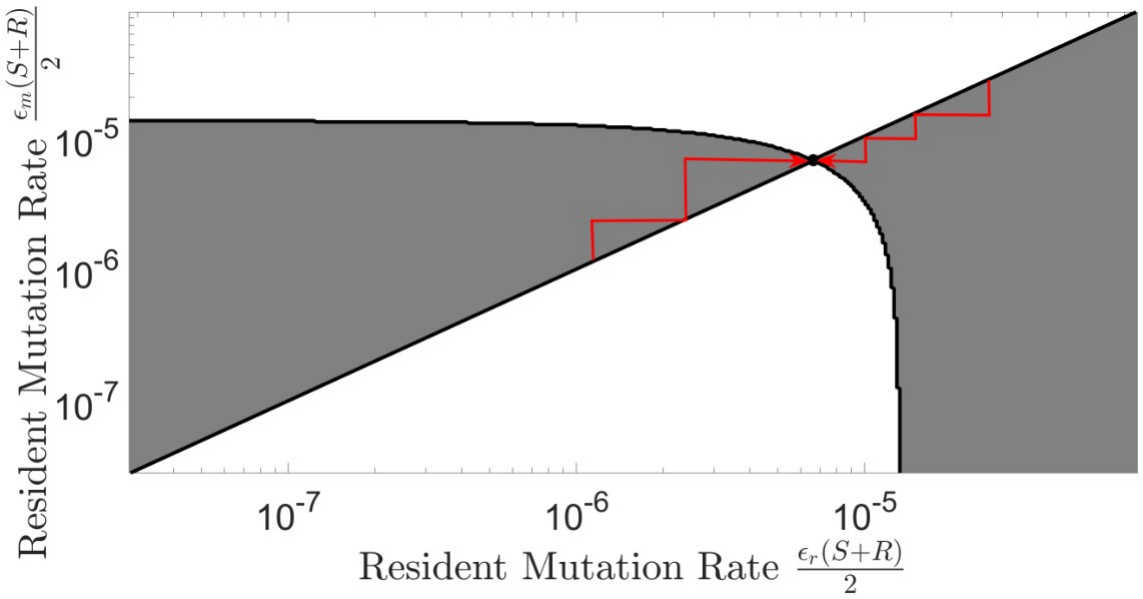
Determination of the optimal switching rates for simple switching between phage- resistant and susceptible states. Implementation of the adaptive dynamics framework to construct a PIP for the simple switching scenario in the presence of both phage and counter-selection (*σ* = 0.05). PIPs show the outcome of any given rare strain, of distinct switching rates, invading a resident system and can be also used to verify the stability of any evolutionary singular points [62]. All parameter values are as given in Table 1. The grey regions represent a successful invasion and the white regions represent a failed invasion. The evolutionary singular strategy is an evolutionary attractor existing at the intersection of these two regions, depicted by the filled black circle. The red arrows are examples of possible evolutionary trajectories, these trajectories alone usually are sufficient in locating the nearest evolutionary attractor.

**Fig 4:**
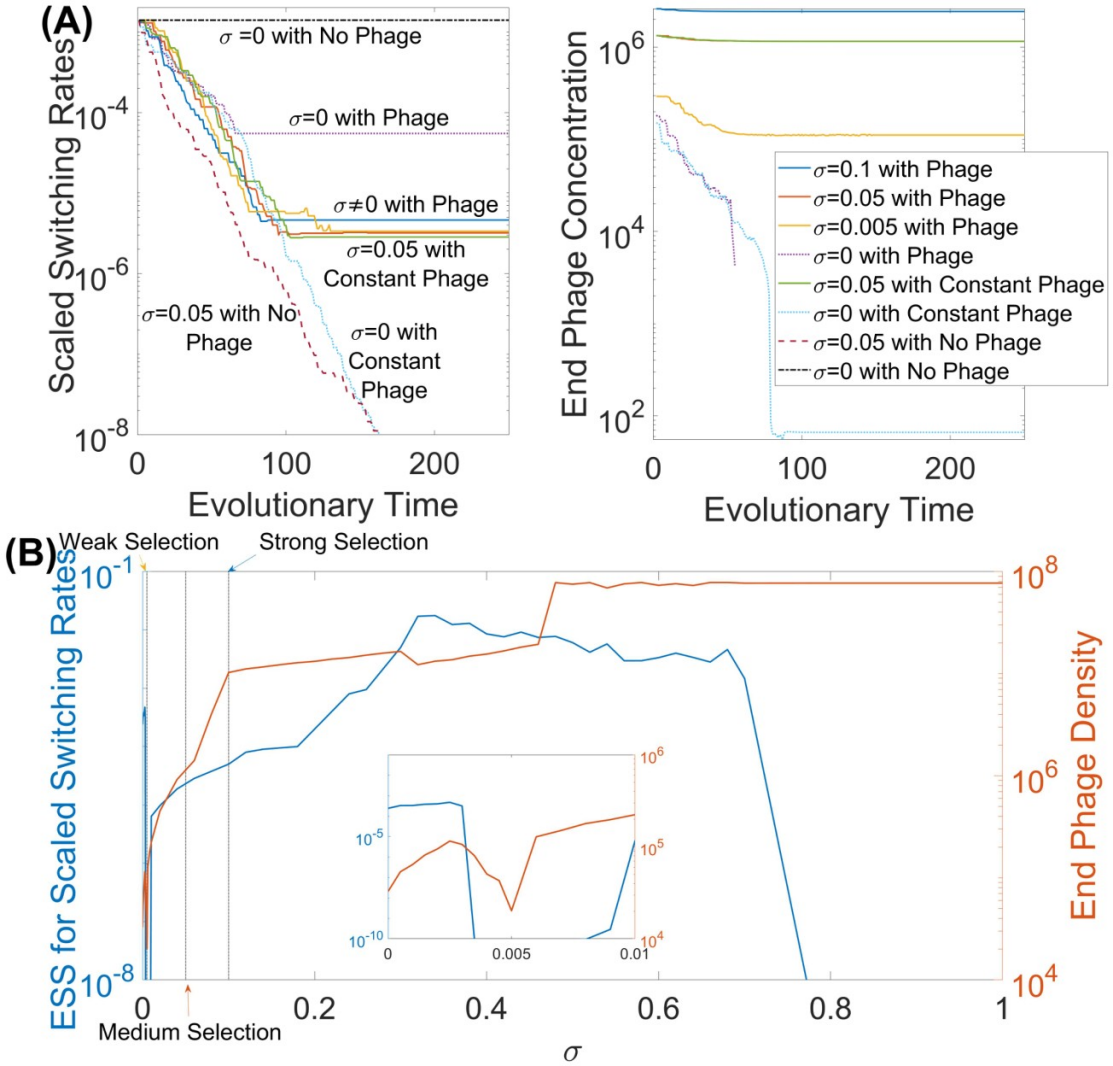
Effect of counter-selection on the ESS. The adaptive dynamics framework was implemented to determine the optimal magnitude of mutation rates *ϵ* for the scenario of simple switching between resistant and susceptible states in the presence of phage and a range of counter-selective pressures. (A) Examples of evolutionary trajectories for different selective pressure scenarios, with the left panel showing the scaled switching rates (*ϵ*(*R* + *S*)/2) values and the right panel showing the corresponding phage densities at the stationary states. Here a single evolutionary time step represents a new invasion. (B) The ESS mutation rates ϵ*(*R* + *S*)/2 and phage densities for varying values of counter-selection, *σ*. The blue curve shows how the ESS mutation rate varies while the orange curve shows the corresponding phage densities at the stationary state. Unless specified otherwise, all parameter values are as given in Table 1.

To explore the evolution of the switching rates, we implemented an adaptive dynamics framework (with the evolutionary parameter *ϵ*). We use Pairwise Invasibility Plots (PIPs). The corresponding PIP, for the environment with both types of selective pressures, is shown in Fig 3 (we omit the PIPs for all other scenarios and environments for the sake of brevity) from which we deduced that this singular point is, in fact, both convergent and evolutionary stable, meaning it is possible to reach an Evolutionarily Stable Strategy (ESS) switching rate ϵ*. These ESS rates can be reached by the evolution of switching rates via invasions and substitutions. We comprehensively explored evolutionary behaviour for the simple switching model with and without phage and with varying counter-selection against the phage resistant state. Fig 4A shows examples of evolutionary trajectories for the magnitude of the mutation rates *ϵ* (with the initial magnitude of switching rates set to be *ϵ* = 1, meaning mutation rates being equal to *R* and *S* as given in Table 1) along with the corresponding phage densities at the end of each simulation.

We also consider the environment with no phage or fitness reduction (i.e. *σ* = 0) and, consequently, no selection (black curve—*σ* = 0 with no phage—in Fig 4A, left panel). For this environment, we observe that the mutation rates do not evolve at all, regardless of the evolutionary starting point. This occurs since no bacterial strains suffer selective pressures and, hence all bacteria have the same fitness, indicating mutation rates will have no impact on the fitness of the overall bacterial population. Adding selection against susceptible bacteria, through the introduction of phage into the system (dotted purple curve—*σ* = 0 with phage—in Fig 4A), has some impact, forcing the mutation rate *ϵ* to evolve to a 10-fold lower value and locking the bacteria into the resistant state. At this point, in the absence of counter-selection against the resistant state, we have the extinction of the phage and halting of evolution (Fig 4A, right panel). To test the effect of maintaining phage selection, we introduced repeated (every 100 hours) exposure to the phage (green curve—*σ* = 0.05 with constant phage—in Fig 4A). This results in the mutation rates *ϵ* evolving below the basal rate and locking the bacteria into the susceptible state. A similar result was observed with counter-selection against the resistant bacteria in the absence of phage, (light blue curve—*σ* = 0 with constant phage—in Fig 4A). The next step was to evaluate the impact of different strengths of counter-selection, implemented as reductions in growth rate, against the phage-resistant bacteria in combination with phage. We started by considering three different values of *σ* (blue, orange and yellow curves—*σ* ≠ 0 with phage—in Fig 4A). For all three cases, the bacteria evolve to have a scaled switching rate of around 10^−5^ with the value of *σ* having very little impact on the ESS value but some effect on phage density with a lower *σ* resulting in a lower phage density.

Variation of *σ* affects the ESS values of switching between resistant and susceptible bacteria. Fig 4B shows that even with very low *σ* values (i.e. starting from 0.005) and thus very low counter-selection pressures against resistant bacteria, there is the coexistence of both the bacteria and phage. This result differs from the well-known scenario with no counter-selection (*σ* = 0), where the phage cannot persist suggesting that counter-selection maintains the phage. Increasing *σ* further results in a gradual increase in the evolutionary stable mutation rates and, as a consequence, an increase in phage density due to the selection trade-off. This increase continues up to a threshold value of *σ* from where there is a rapid evolution of mutation rates to below the basal rate. Thus when counter-selection against the phage-resistant bacteria becomes very high, remaining in the phage-susceptible state for bacteria is more beneficial than switching between states.

### 3.2 Scenario 2: Single gene PV

**Fig 5:**
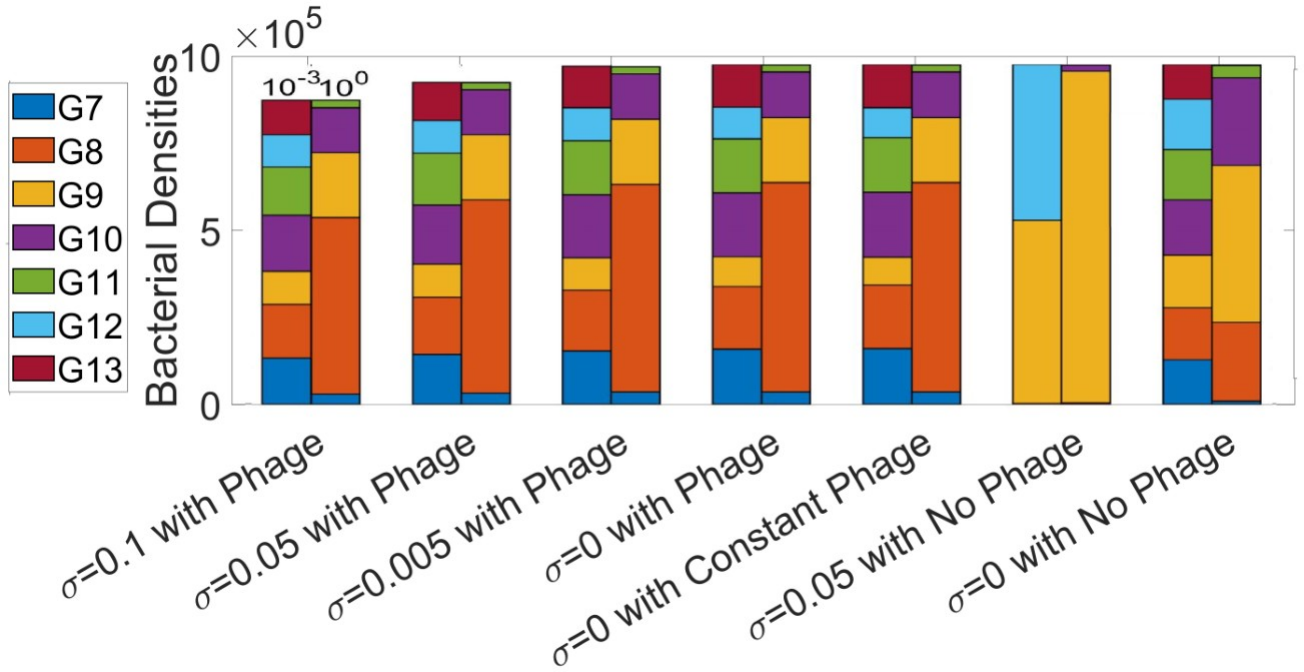
Tract length distributions at the stationary states for varying environments and evolvable switching rates for PV occurring in a single gene. The right bars correspond to the switching rates exactly as in Table 2 (*ϵ* = 1) and the left bars correspond to switching rates *ϵ* = 10^−3^ smaller than the switching rates given in Table 2. All parameter values are as given by Table 1 and the phage-susceptible states are associated with tract lengths G9 and G12 whereas counter-selection, where applied, is imposed on all other tract lengths.

This scenario considers a single PV locus capable of mutating between seven possible polyG/C repeat numbers, of which two (9 and 12) are associated with gene expression and the phage-susceptible state. Before investigating the evolution of the switching rates, we compared the distribution of bacteria with each repeat number for the experimental mutation rates (as shown in Table 2 and with *ϵ* = 1) with a 1,000-fold lower rate at the stationary state (*ϵ* = 0.001) (Fig 5). We need this information to understand how a decrease in mutation rates would affect the distribution of bacteria across the strains.

In the absence of phage and counter-selection, the stationary phase population for the experimental mutation rates exhibited a bias towards 8,9 and 10 repeats (53.95% in the OFF state) whereas, with the lower mutation rate, there was an even distribution between all repeat numbers. A similar pattern was observed when the phage was introduced with or without counter-selection apart from a slight increase in the proportion of the population having a G8 tract (the OFF phage-resistant state) for the experimental mutation rates and hence an overall shift to a higher proportion of the OFF state (79.89% with medium counter-selection and 80.93% without counter-selection for the experimental mutation rates). As the lower mutation had a similar OFF proportion without phage, this explains why the introduction of phage did not change the repeat tract distribution. The only scenario with a notable difference was growth reducing counter-selection in the absence of phage (note that this selection acts against the OFF gene expression states). With the lower mutation rate, there is a similar proportion of the G9 and G12 tracts whereas, for the higher mutation rate, the population is strongly shifted to G9. In both cases, there is a very high ON state proportion (97.78% for the experimental mutation rates and 99.99% for the lower mutation rate). Thus the bacteria are locked into the susceptible state, a logical outcome as the only selective pressure was acting on the resistant state. So although the ON/OFF distributions are very similar the tract length distributions can be quite different, which highlights how a change in the mutation rate can have significant effects on the resulting stationary state. These results suggested that when accounting for PV in a single locus, the bacteria are very sensitive to the strength of counter-selection.

**Fig 6:**
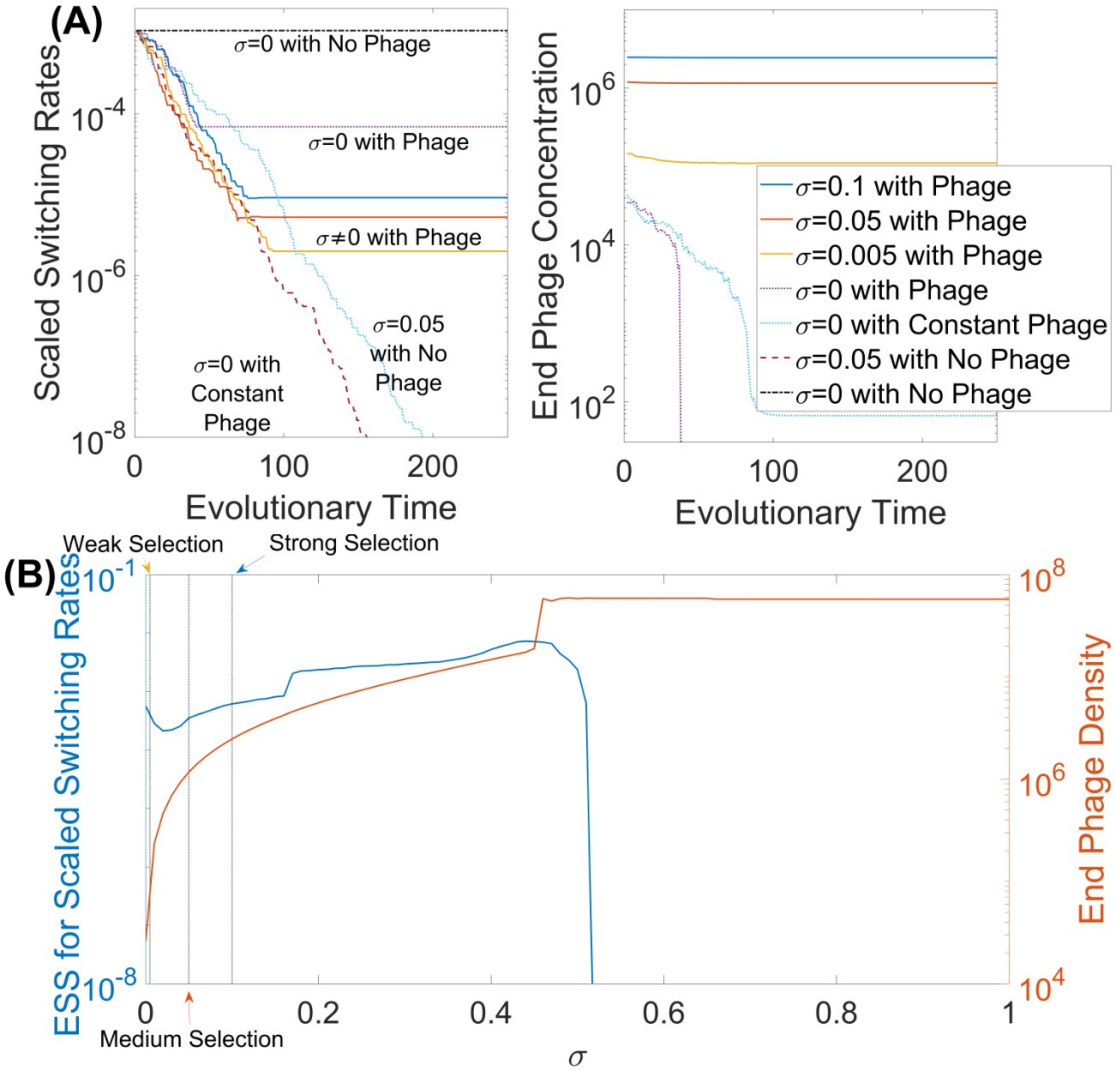
Determination of the optimal mutation rates of a single PV gene subject to phage-mediated selection and varying levels of counter-selection with an adaptive dynamics framework. (A) Examples of evolutionary trajectories for different selective pressure scenarios, with the left panel showing the scaled switching rates (given by *ϵ*(∑(*α* + *β*))/14) and the right panel showing the corresponding phage densities at the stationary states. (B) Effect of varying values of counter-selection, *σ*. The blue curve shows how the ESS mutation rate varies with the orange curve showing the corresponding phage densities at the stationary state. Unless specified otherwise, all parameter values are as given in Table 1.

We explore the evolutionary outcomes for the single PV locus model with and without phage and varying counter-selection acting against the phage resistant states. Fig 6A displays examples of evolutionary trajectories of *ϵ* (along with the corresponding phage densities at the end of each simulation). We also vary the strength of the counter-selection *σ* with the dependence shown in Fig 6B. All of the evolutionary trajectories result in ESS values very close to that of scenario 1, as expected considering that both scenarios involve a single locus. A key difference between the results of the two scenarios is that varying the strength of counter-selection results in a greater impact on the ESS with the selection cut-off (the point at which counter-selection is so strong that the bacteria evolve below the basal rate) occurring at a much lower value of *σ*. This significant reduction in cut-off may be explained by the difference in some switching rates due to the inclusion of evolvable switching to lower repeat numbers, for example, the G7-G8 switching rates are almost ten-fold lower than the rates in the first scenario.

### 3.3 Scenario 3: Two genes PV

**Fig 7:**
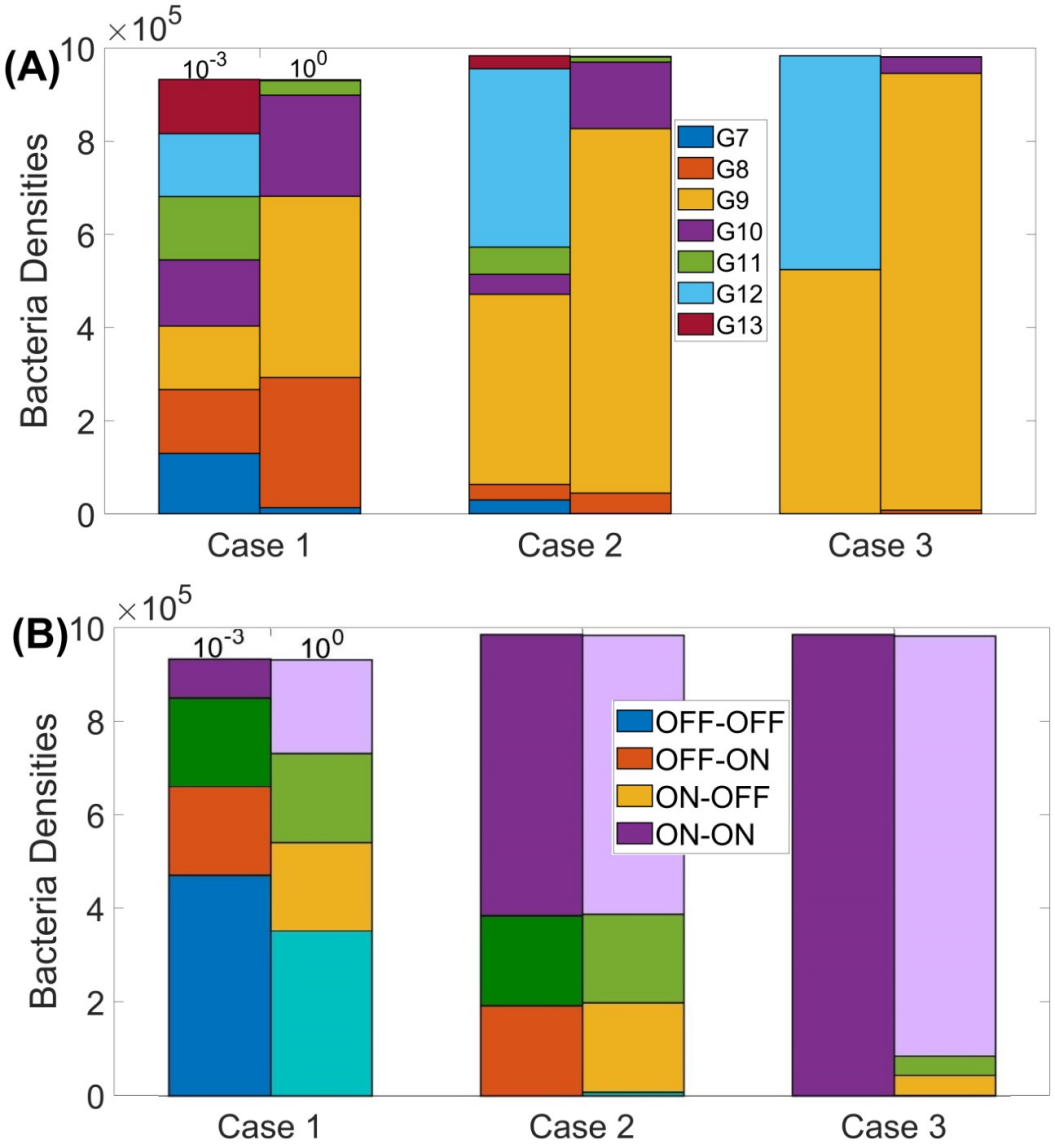
Repeat number and expression state distributions at the stationary states for PV occurring in two genes in the presence of phage and with three differing cases of counter-selection. The phage-susceptible state is the ON-OFF combinations while all other combinations are phage-resistant. Case 1, counter-selection (*σ* = 0.05) on all phage-resistant states; case 2, counter-selection on OFF-OFF state only; case 3, counter-selection on OFF-OFF of *σ* = 0.05 and on ON-OFF/OFF-ON at *σ* = 0.025. Note that the ON-ON state (G9-G9, G9-G12, G12-G9 and G12-G12) are not subject to any counter-selection in scenarios 2 and 3. (A) Distributions of repeat numbers for the first PV gene at the stationary state. (B) Corresponding state distributions at the stationary states, with ON-OFF being phage-susceptible. In both figures, the right bars correspond to the switching rates exactly as in Table 2 (*ϵ* = 1) and the left bars correspond to switching rates 10^−3^ smaller than the switching rates given in Table 2 (*ϵ* = 0.001). All other parameter values are as given by Table 1.

The final scenario explores two phase-variable loci, each with seven possible poly G/C repeat numbers. Here only the ON-OFF state is susceptible to phage infection. This mimics the real-world situation for the F336 phage infecting *C. jejuni* strain NCTC11168 where the first gene, *cj1421*, synthesises the phage receptor while switching ON of a second gene, *cj1422*, results in the synthesis of an epitope that blocks adhesion. We then consider three different possible cases of counter-selection (*σ*). (outlined in Section 2.5). For the first case, we choose ON-OFF to have *σ* = 0 with all others characterised by the same *σ*. Secondly, we consider OFF-OFF to have a nonzero *σ*, with all others being *σ* = 0. For the third and final case, we choose ON-ON having *σ* = 0, OFF-OFF with a nonzero *σ*, with ON-OFF and OFF-ON having half the *σ* value as OFF-OFF. Before investigating the evolution of switching rates for each of these cases, we compare the distribution of bacteria with each repeat number for the first locus and the state distributions for the experimental mutation rates (as shown in Table 2 and with *ϵ* = 1) with a 1,000-fold lower rate at the stationary state (*ϵ* = 0.001) (Fig 7).

In case 1, where we have counter-selection acting on all phage resistant states, we observed an even balance between tracts of 9 (the ON state) and 8/10 (OFF states) and between the three phage-resistant states. For cases 2 and 3 there is a strong bias towards tracts of 9 and to the ON-ON state (i.e. those with the lowest level of counter-selection). Thus the bacteria are locked into a phage-resistant state, which suggests that PV of these genes could only evolve if there is another external selective pressure acting on these genes when both are in the ON state. For all three cases, there is a difference between the tract length distributions, this is due to the bacteria switching to the strains with the least selection acting on them at a slower rate, for the lower mutation rate, meaning bacteria get locked into a wider range of states.

**Fig 8:**
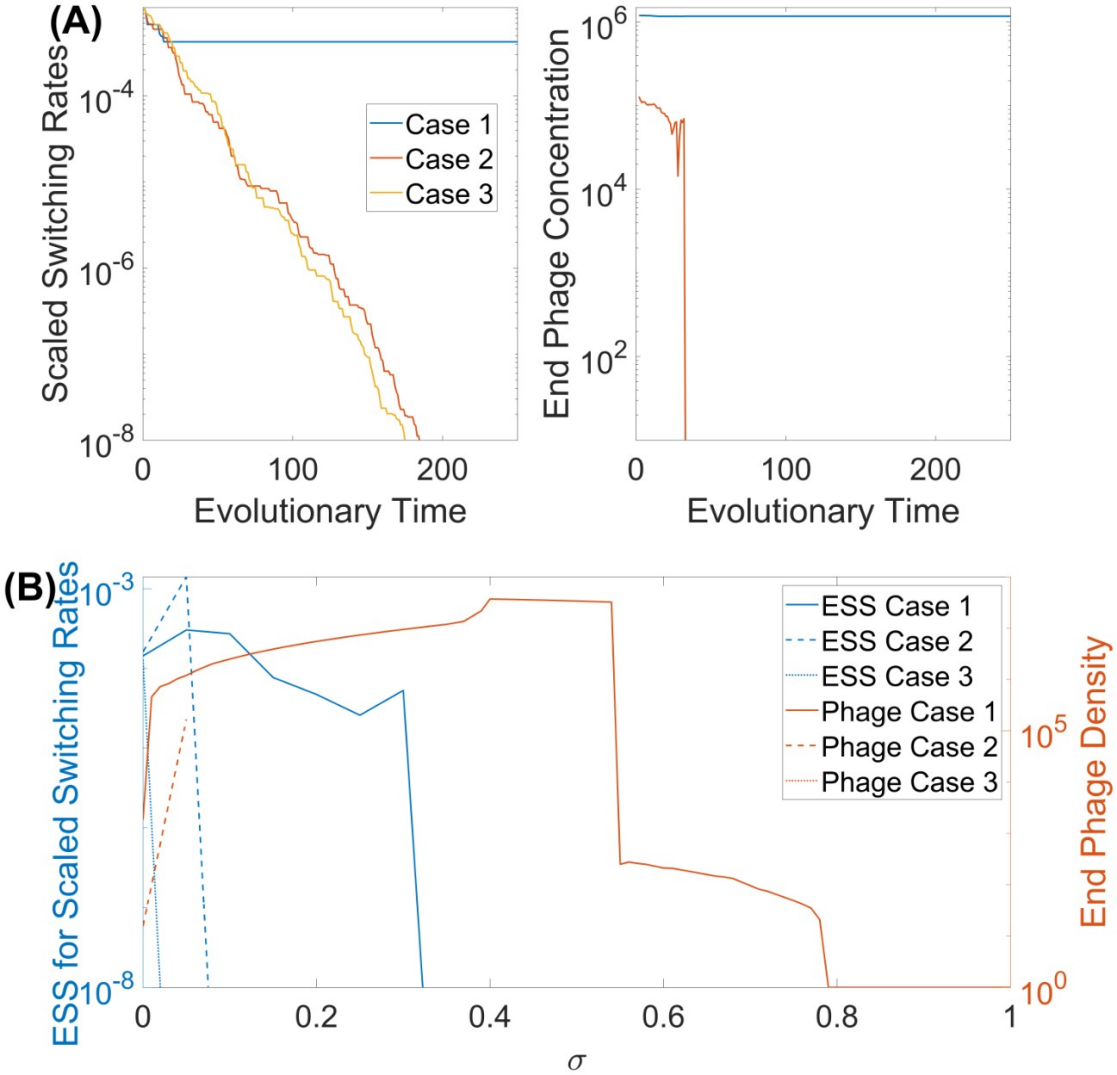
Implementation of the adaptive dynamics framework for the scenario with two single genes being simultaneously subjected to PV to determine the optimal mutation rates of bacteria for three different counter-selection cases. (A) Examples of evolutionary trajectories, with the left panel showing the scaled switching rates (*ϵ*(∑(*α* + *β*))/14) and the right panel showing the corresponding phage densities at the stationary states. (B) Implementation of the adaptive dynamics framework for varying values of counter-selection, *σ*. The blue curve shows how the ESS mutation rate varies with the orange curve showing the corresponding phage densities at the stationary state. Unless specified otherwise, all parameter values are as given in Table 1.

Evolution of the mutation rates was explored for each of the three selective mechanisms with both counter-selection (*σ* = 0.05) and the presence of phage. Fig 8A shows examples of evolutionary trajectories of the magnitude of mutation rates along with the corresponding phage densities. For case 1 (blue curve), where counter-selection acts on all resistant states, there is a limited evolution of the mutation rate and a very rapid approach to an ESS with stable maintenance of phage. Contrastingly, in cases 2 and 3 (orange and yellow curves, respectively), there is no phage persistence and this results in the mutation rates evolving below the basal rates fixing the bacteria into the ON-ON state, the state with the lowest level of selection.

The ESS for case 1 is close to the experimental values (see Table 2), which indicates that the model is a good mimic of the actual biological situation for *C. jejuni*. However, our understanding is that counter-selection favours the ON-ON phage-resistant state which results in a low ESS as shown for cases 2 and 3. We interpret this finding as evidence for another selective pressure acting specifically on the ON-ON state but not the ON-OFF state. In Fig 8B, we examined the impact of counter-selection strength, *σ*. Case 1 tolerates counter-selection up to 0.33 before the ESS evolves to very low rates. Cases 2 and 3 are much more sensitive to the strength of counter-selection due in part to the rapid elimination of phage as counter-selection increases.

## 4 Discussion

This theoretical study focuses on building a deeper understanding of the influence of PV on bacteria-phage interactions and the long-term evolution of hypermutable mechanisms of phage resistance. These phenomena are widespread among bacteria and have significant differences to phage resistance that arises by a combination of random mutations and frequency-dependent selection. Additionally, our models are built on a specific system utilising experimentally-derived repeat-driven PV rates for *C. jejuni* and predation by the F336 phage. Thus, our findings have both direct biological relevance but also allow for derivation of general principles.

### 4.1 Distinguishing features and generic findings of our PV-phage models

Reflecting known biological attributes, mathematical models of PV have some particularities as compared to those dealing with random mutations. These include; very high mutation rates per cell division, reversibility of mutations (this means the ratio of insertion and deletion probabilities is around 10, see Table 2), complex architecture of mutation networks (transition is only possible between particular states and not between all of them), and divergent costs of bacterial resistance (counter-selection) for different states or combinations of states. Note, for example, that in the case of random mutations, the ratio between direct and inverse mutation probabilities can be as large as 10^6^ or 10^7^ [41].

Another key aspect of PV is that all variants (e.g. phage resistant and susceptible states) and tracts lengths can be rapidly generated from a single cell due to the high mutation rates. This means that these variants can be rapidly recovered following exposure of the population to narrow bottlenecks and contrasts with other mutational mechanisms that become fixed into a specific state in this scenario. For our models, outcomes are likely to be independent of initial conditions and therefore immune to loss of biodiversity as a result of exposure to narrow bottlenecks. We show (see Figs A and B in S1 Text) that results are independent of initial bacterial densities as an indication of how our model output differs to a random mutational mechanism and frequency-dependent switching.

Our models use a framework combining population dynamics and evolutionary game theory. Although some previous studies investigated aspects of phage-bacteria co-evolution [13, 33] or phage therapy [34–36], very few have focused on the evolution of bacterial mutation rates and PV switching between resistant-susceptible states under realistic assumptions such as a two loci PV system [63–65]. Utilising experimentally-derived PV rates for *C. jejuni* genes, we compare three different PV scenarios in a theoretical model wherein we apply realistic assumptions on the phage infection parameters and counter-selection values for the phage-resistant state(s).

From these models we derive a series of generic insights. Firstly in all models (starting from the simplest one), we observe that the mutation rate evolves to some intermediate values, which indicates that very high and very low mutation rates are evolutionarily disadvantageous. Secondly, the evolution of the phase-variable resistance mechanism requires continual exposure to the phage and to counter-selection against the phage-resistant state (we discuss in the next section the quantitative aspects of this interaction). Thirdly, counter-selection acts on specific phage-resistant states (not necessarily all) and hence two-loci PV systems can behave in contrasting ways to single-locus PV systems as well as to the simple R-S model, where phages can only persist if there is counter-selection against the phage-resistant state. We reflect below on some of the biological relevance and applications arising from both these generic and more specific insights of our modelling.

### 4.2 Determinants of the emergence of evolutionary stable PV switching rates

Applying advanced evolutionary modelling techniques to our phase-variable system led to the detection of evolutionarily stable mutational switching rates (ESS) for all mutational settings when both phage and the growth debilitating counter-selection were present. This result is of general significance as although parameter values were based on realistic estimates of biological parameters for *C. jejuni* and the F336 phage, the principles and modelling frameworks apply to other bacterial species where PV mediates phage resistance. In the case with both forms of selection, phage and the growth reducing counter-selection (*σ* > 0), we observe rapid evolution away from the high mutation rates (i.e. decrease in *ϵ*). High mutation rates would signify replenishment of the pool of susceptible bacteria which, in turn, should increase the abundance of phage, which would not be beneficial for the bacterial population. Less evident is the fact that mutation rates in the model do not continue to decrease indefinitely (approaching zero) but instead their evolution stops at some intermediate value ϵ* (which we define as an ESS). Critically, we only observe PV rates comparable to observed rates (10^−3^) in the presence of phage and when counter-selection was within a specific narrow range (i.e. 0.3 to 0.7; Fig 4B).

To better understand this result, we studied the temporal dynamics with both types of selection (phage and the growth-reducing counter-selection) at low mutation rates, when *ϵ* < ϵ*. These temporal dynamics showed bacterial and phage coexistence, even at very low mutation rates, furthermore, the ratios of resistant and susceptible variants are very close to those observed at the ESS. However, our observations show that at low switching rates, the bacterial densities are unable to quickly adapt to changes in the environment (note that the population densities exhibit small oscillations around the equilibrium states). Therefore, when a bacterial strain competes with another one possessing a higher mutation rate *ϵ* and therefore more frequent switching, the lower mutating strain will be eventually outcompeted by the faster mutating strain before it has a chance to adapt to the competing environment. Thus there is a limit to the trend towards the evolution of low mutations. Conversely, high counter-selection against the phage-resistant states favours the low mutating strain as switching to the phage-resistant state is not advantageous and hence there is an upper limit on the evolution of high mutation rates.

From the biological point of view, it’s important to note that whilst previous observations included; short-lived benefits from genomewide mutators during co-evolution with phages [66], or phage extinction due to the evolution of mutator phenotypes with or without co-selection from sub-inhibitory antibiotic concentrations [35, 67], our observations are a novel example of the potent impact of phages on the evolution of localised hypermutation in phage interactive genes.

### 4.3 Two Genes PV—Experimental values

An under-explored area of PV biology is how reversible mutability can evolve in multiple loci. Resistance to phage F336 in *C. jejuni* strain NCTC1168 was known to strongly depend on two phase-variable loci with evidence that counter-selection could act on these loci [21]. We explored this scenario using a model with two loci, a phage requiring one of the four possible expression combinations for adhesion and three different cases of growth-limiting counter-selection (Section 3.3). For case 1, where counter-selection acts only on the phage resistant states (and susceptible states can grow are full capacity), the ESS rates were very close to the experimental switching rates displayed in Table 2. Strongly suggesting that our computational model has the potential to closely match experimental settings and could effectively be used to mimic the behaviour of the *C. jejuni* bacteria. Meaning the model is potentially beneficial in designing experiments to test the effects of phage on bacterial populations in natural interactions. However, case 1 is predicated on counter-selection by serum components only acting when there is a single phosphoramidite residue attached to a specific position on the capsule, whereas it is more likely that the counter-selection is blind to the attachment site and is affected by the number of phosphoramidite residues. When these scenarios were tested (cases 2 and 3), we observed that experimental mutation rates resulted in the elimination of susceptible bacteria and locking of the bacteria into the resistant state as the switching rates evolved below the basal rate. In this case, phage therapy using the considered single phage type should simply fail. This observation leads to the suggestion of an alternate scenario where another type of phage can attach and infect *C. jejuni* when both genes are in the ON state. There has been some work with computationally modelling a system with two distinct phages, so this could prove to be an interesting extension to our research [41].

### 4.4 Constant phage influx

In most simulations, we consider a single exposure of bacteria to phage, however, we briefly investigate the influence of a constant influx of phage into our system as is likely to occur in nature due to the phage replicating in a sub-set of multiple discrete populations. Considering the example of medium counter-selection with phage (Fig 4), ordinarily, we have the presence of phage at the ESS, adding a regular phage influx does not impact the phage density at the ESS nor the value of the ESS itself. Contrastingly, no counter-selection with a single influx of phage results in the extinction of phage and rapid approach to a high ESS rate, however, with constant phage influx the ESS is forced below the basal rate, locking the bacteria into the phage resistant state. Together these results suggest that in a system where the phage persists at the ESS, the constant influx does not impact the overall evolution, however, if we have phage extinction, adding constant influx results in further evolution past the basal rate. We reiterate this result in the two gene PV scenario (see Fig C in S1 Text for detail), particularly cases 2 and 3, where a constant phage influx results in bacteria locking into a fixed ON-ON state at a much faster rate than when subjected to only one phage exposure. In summary, our model shows that phages may be critical drivers of the evolution of PV switching rates.

### 4.5 Application of our findings to phage therapy to efficiently reduce the presence of *C. jejuni* in chicken meat

Our theoretical model predicts potential inefficiencies for phage therapy when it is applied to live chickens with ongoing replication of pathogenic bacteria with a phase-variable phage-resistance mechanism. In the absence of phages, in live birds, where the only selective pressure is the growth-reducing one that acts only on the phage resistant states, the bacterial population will be driven into a majority phage susceptible state (Fig 2 shows that for the simple PV model there is an 6-fold more susceptible than resistant bacteria in the absence of phage). Introduction of phage into this system will result in an initial loss of bacteria, due to the majority of them being susceptible to phage, but will be followed by a switch to a phage-resistant state with rapid recovery of the initial bacterial concentration. On the contrary, introducing phage into this scenario with no ongoing bacterial replication (e.g. by treating chicken carcasses) will result in an almost 10-fold reduction in the total bacterial load.

By modelling the realistic scenario of two PV loci, we found that the treatment of live chickens with an F336-like phage can only be considered in the case where it is known that counter-selection acts only on the phage resistant states of the *C.jejuni* strain. In this case, a balance between susceptible and phage-resistant states is possible, and hence treatment may reduce the number of phage-susceptible bacterial cells. In the other cases, characterised by a strong bias towards tracts of 9 and to the ON-ON state, most bacterial cells will be in a F336-phage-resistant state and hence treatment with the F336 phage alone would be ineffective. These results show that careful evaluation of the drivers of oscillation of PV states are required where phages that target phase-variable epitopes will be utilised in phage therapy.

### 4.6 Future work

Future work will need to focus on determining the nature and strength of counter-selection acting on the F336 phage states and whether other phages bind to the F336-phage resistant state. This experimental data will facilitate improved modelling of the evolution of PV in *C. jejuni*. Palmer *et. al*. (2013) [63] considered bacteria in switching environments, with the selective pressure switching between phage and counter-selection with each change of environment. Other scenarios are for intermittent and unpredictable exposure to phages and imposition of frequent non-selective bottlenecks. Exploring these more complex modelling frameworks could lead to a viable explanation of the divergence between our modelling results and the experimentally observed switching rates.

## Supporting information

S1 Text Fig A. Substrate, bacterial and phage densities in the absence of PV with varying initial starting densities. Fig B. Substrate, bacterial and phage densities in the presence of PV with varying initial starting densities. Fig C. Scenario 3 with Constant Influx of Phage. Implementation of the adaptive dynamics framework for the scenario with two single genes being simultaneously subjected to PV to determine the optimal mutation rates of bacteria for three different counter-selection cases with a constant influx of phage.
(PDF)
