# Supplementary material for: How does feedback from phage infections influence the evolution of phase variation in Campylobacter?
Source: PLoS Comput Biol. 2021 Jun 14;17(6):e1009067. doi: 10.1371/journal.pcbi.1009067 (PMC8224891; doi:10.1371/journal.pcbi.1009067)
Supplement: S1 Text — Fig A. Substrate, bacterial and phage densities in the absence of PV with varying initial starting densities. Fig B. Substrate, bacterial and phage densities in the presence of PV with varying initial starting densities. Fig C. Scenario 3 with Constant Influx of Phage. Implementation of the adaptive dynamics framework for the scenario with two single genes being simultaneously subjected to PV to determine the optimal mutation rates of bacteria for three different counter-selection cases with a constant influx of phage. (PDF) [file pcbi.1009067.s001.pdf]

Supplementary File : How Does Feedback from Phage Infections Influence the Evolution of Phase Variation in *Campylobacter*?

Simran K. Sandhu[1], Christopher D. Bayliss[2], Andrew Yu. Morozov[1,3*]

1. Department of Mathematics, University of Leicester, Leicester, UK
2. Department of Genetics and Genome Biology, University of Leicester, Leicester, UK
3. Institute of Ecology and Evolution, Russian Academy of Sciences, Moscow, Russia

S1 A Independence of Initial Conditions

An important aspect of the study is allowing for reversible mutational switching, which is different from some of the other well-known mechanisms such as frequency-dependent switching. Modelling mutations through Phase Variation (PV) shows that the resultant bacterial densities become independent of initial conditions. An insightful example is shown in the two following figures (Fig. A and B and further details are provided below). This signifies that the exposure of the PV bacterial population to a very narrow bottleneck (which is a frequent biological phenomenon) does not prevent the very rapid emergence of resistant and sensitive variants in all potential combinations. This is significantly different to other mutational mechanisms and frequency-dependent selection as in these cases narrow bottlenecks fix the population into one state (e.g. susceptible or resistant). Although further investigation of bottlenecks is beyond the scope of this current study, our preliminary results indicate the independence of initial conditions for the PV mechanism and demonstrate how these mechanisms are divergent from other resistance phenomena.

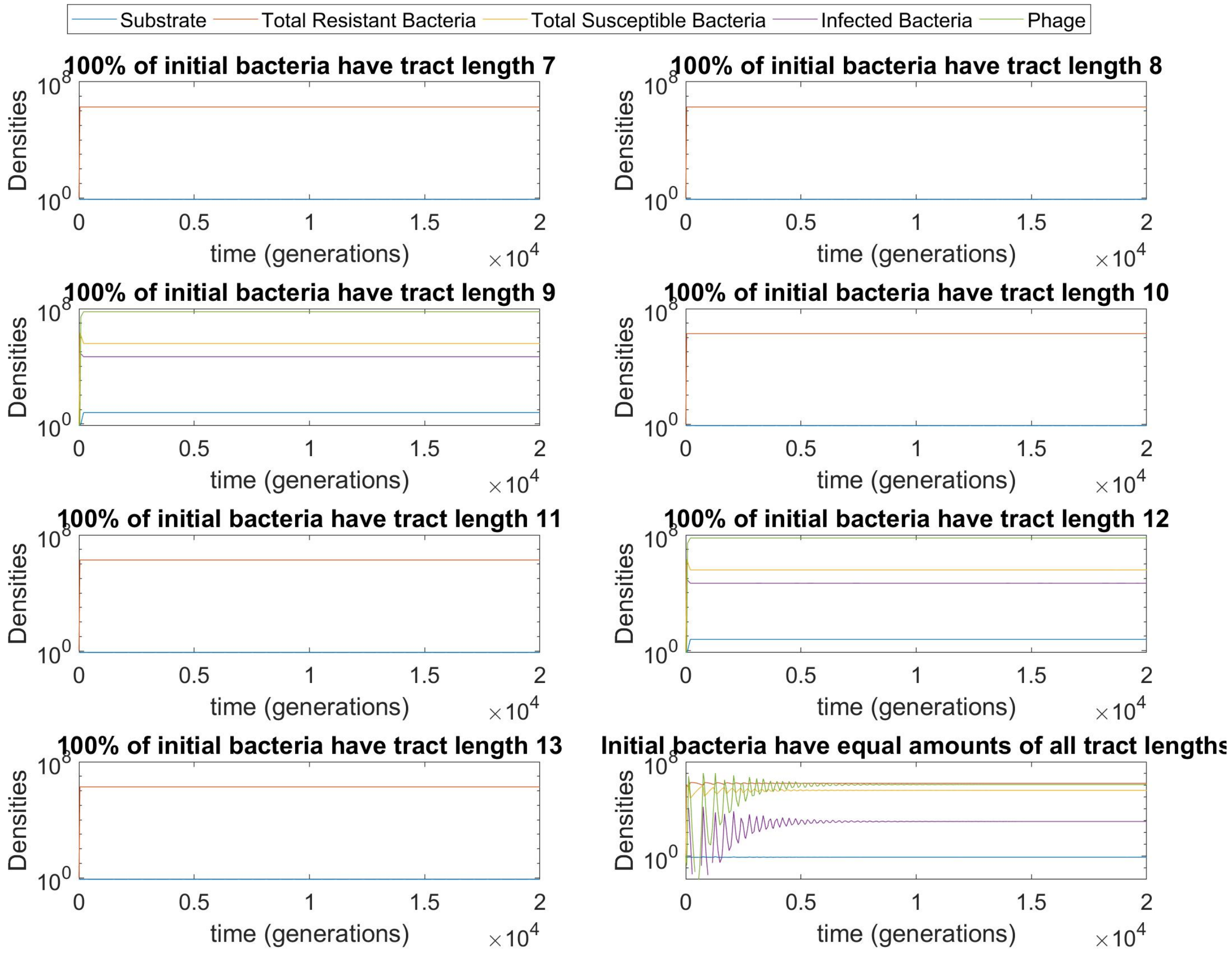

Fig. A: Substrate, bacterial and phage densities in the absence of PV with varying initial starting densities. The initial starting density of total bacteria is $1CFU/ml$ and it's make up is defined by each plot title, the starting density of substrate is 0.8691 and the initial phage density is $1PFU/ml$. The model is described by equations (1-5) with counter-selection ($\sigma = 0.05$). All other parameter values are taken from Table 1.

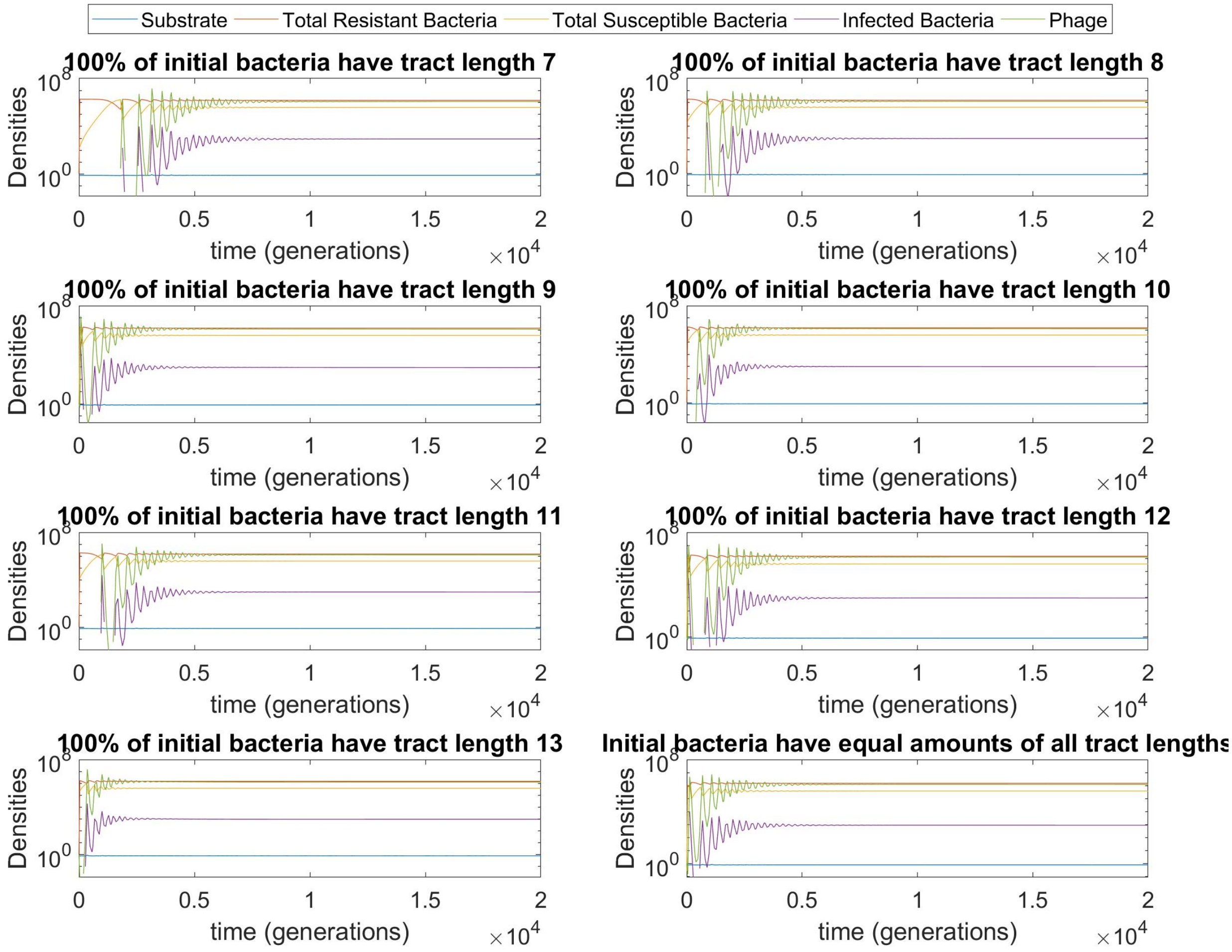

Fig. B: Substrate, bacterial and phage densities in the presence of PV with varying initial starting densities. The initial starting density of total bacteria is $1CFU/ml$ and it's make up is defined by each plot title, the starting density of substrate is $0.8691\mu g/ml$ and the initial phage density is $1PFU/ml$. The model is described by equations (1-5) with counter-selection ($\sigma = 0.05$) and involves a mutation strategy of PV occurring in a single gene at rates given in Table 2 (i.e. the scaling factor ϵ is set to 1). All other parameter values are taken from Table 1.

In Fig. A, in the absence of PV (i.e. $\epsilon = 0$), it is clear that the resulting densities are highly dependent on the initial starting densities. On the other hand, Fig. B shows that in the presence of PV (i.e. $\epsilon = 1$), the results are independent to these initial densities, with the system reaching the same stationary state regardless of the starting point.

S1 B Scenario 3 with Constant Influx of Phage

The final scenario considered two phase-variable loci each with seven possible poly G/C repeat numbers. Within the main text we presented results with a single influx of phage, however, it is mentioned in the main text that these results do not change if instead a constant influx of phage is considered. Here we present the corresponding results. The adaptive dynamics framework was implemented for each of the three selective mechanisms in the environment with both counter selection ($\sigma = 0.05$) and the constant influx of phage. The figure shows examples of evolutionary trajectories of the magnitude of mutation rates along with the corresponding phage densities.

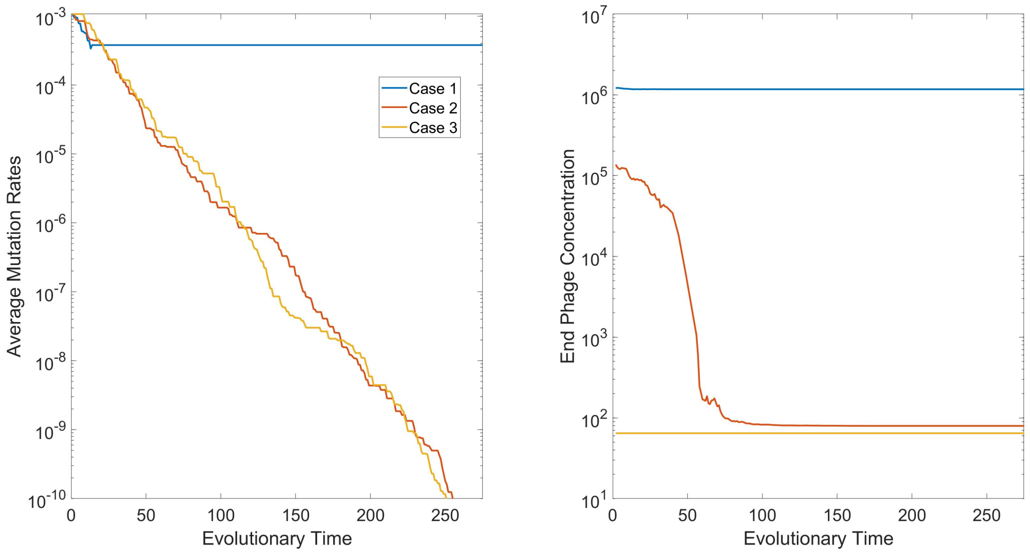

Fig. C: Implementation of the adaptive dynamics framework for the scenario with two single genes being simultaneously subjected to PV to determine the optimal mutation rates of bacteria for three different selection cases with a constant influx of phage. The left panel shows the ESS values and the right panel shows the corresponding phage densities at the stationary states. Unless specified otherwise, all parameter values are as given in Table 1.
